# Diagnostic value of bronchoalveolar lavage fluid metagenomic next-generation sequencing in pediatric pneumonia

**DOI:** 10.3389/fcimb.2022.950531

**Published:** 2022-10-27

**Authors:** Wenhua Deng, Huan Xu, Yabin Wu, Jie Li

**Affiliations:** ^1^ Pediatric Respiratory Department, Maternal and Child Health Hospital of Hubei Province, Wuhan, China; ^2^ Department of Scientific Affairs, Vision Medicals Center for Infection Diseases, Guangzhou, China

**Keywords:** metagenomic next-generation sequencing (mNGS), conventional microbiological tests (CMTs), bronchoalveolar lavage fluid (BALF), pediatric, pneumonia

## Abstract

**Objectives:**

The aim of this study was to evaluate the diagnostic value of bronchoalveolar lavage fluid (BALF) metagenomic next-generation sequencing (mNGS) versus conventional microbiological tests (CMTs) for pediatric pneumonia.

**Methods:**

This retrospective observational study enrolled 103 children who were diagnosed with pneumonia and hospitalized at Hubei Maternity and Child Health Care Hospital between 15 October 2020 and 15 February 2022. The pneumonia diagnosis was based on clinical manifestations, lung imaging, and microbiological tests. Pathogens in the lower respiratory tract were detected using CMTs and BALF mNGS (of DNA and RNA). The diagnostic performance of BALF mNGS was compared with that of CMTs.

**Results:**

In 96 patients, pathogens were identified by microbiological tests. The overall pathogen detection rate of mNGS was significantly higher than that of CMTs (91.3% vs. 59.2%, *p* = 0.000). The diagnostic performance of mNGS varied for different pathogens; however, its sensitivity and accuracy for diagnosing bacterial and viral infections were both higher than those of CMTs (*p* = 0.000). For the diagnosis of fungi, the sensitivity of mNGS (87.5%) was higher than that of CMTs (25%); however, its specificity and accuracy were lower than those of CMTs (*p* < 0.01). For the diagnosis of *Mycoplasma pneumoniae*, the specificity (98.8%) and accuracy (88.3%) of mNGS were high; however, its sensitivity (42.1%) was significantly lower than that of CMTs (100%) (*p* = 0.001). In 96 patients with definite pathogens, 52 cases (50.5%) were infected with a single pathogen, while 44 cases (42.7%) had polymicrobial infections. Virus–bacteria and virus–virus co-infections were the most common. *Staphylococcus aureus*, *Haemophilus influenzae*, rhinovirus, cytomegalovirus, parainfluenza virus, and fungi were more likely to be associated with polymicrobial infections.

**Conclusions:**

BALF mNGS improved the detection rate of pediatric pneumonia, especially in mixed infections. The diagnostic performance of BALF mNGS varies according to pathogen type. mNGS can be used to supplement CMTs. A combination of mNGS and CMTs may be the best diagnostic strategy.

## Introduction

The World Health Organization reports that pneumonia is the leading cause worldwide of mortality among children younger than 5 years old (GBD 2015 LRICollaborators, 2017). In clinical practice, identifying pathogens in infectious diseases is a difficult problem. Conventional microbiological tests (CMTs) are limited in their scope for pathogen detection; they are time-consuming, have low detection rates, and usually detect only single pathogens. Although polymerase chain reaction (PCR) tests and serological detection have expanded the detection range of CMTs and increased detection rates, clinicians must first identify the type of pathogen. It is important to diagnose pathogens quickly and accurately in order to shorten the hospital stay and reduce complications and mortality.

Metagenomic next-generation sequencing (mNGS) is an unbiased detection technology that can detect multiple pathogens across a wide range. It is relatively time-saving, with a turnaround time of 24–48 h. mNGS has been shown in recent years to be advantageous and viable for the identification of respiratory tract infection pathogens (Leo et al., 2017). However, sequencing DNA and RNA at the same time using mNGS has rarely been reported. In the present study, we compared the diagnostic value of CMTs and mNGS (DNA and RNA) for detecting pneumonia pathogens in children.

## Methods

### Study design and patient selection

**Figure 1:**
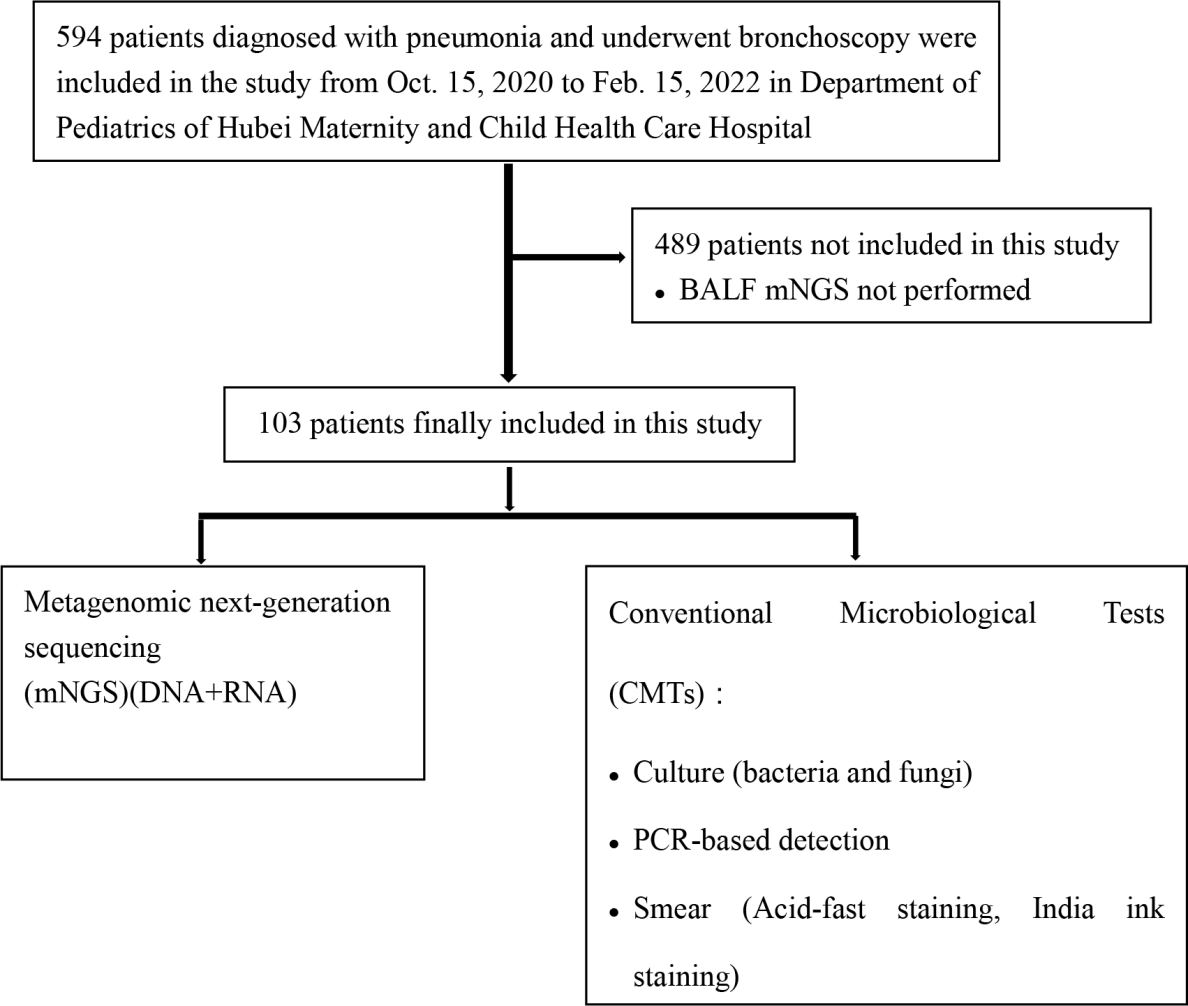
Flow diagram of patient inclusion and exclusion.

This retrospective observational study enrolled children who were diagnosed with pneumonia and hospitalized at the Maternity and Child Health Care Hospital of Hubei Province between 15 October 2020 and 15 February 2022. The inclusion criteria were as follows: (1) the child presented with typical clinical signs of pulmonary infection, such as fever, cough, sputum, and dyspnea; and (2) the diagnosis of pulmonary infection was supported by radiological evidence (e.g., chest computed tomography scan). We excluded patients who were not tested using bronchoalveolar lavage fluid (BALF) mNGS (DNA and RNA). A total of 103 children were enrolled in this study. The recruitment process is illustrated in **Figure 1**. Patient age, sex, symptoms, laboratory findings, lung imaging, bronchoscopic findings, and medical history were recorded. All included patients underwent bronchoscopy to obtain BALF samples for use in CMTs and mNGS. Bronchoscopies were performed by experienced bronchoscopy physicians according to standard safety protocols. No serious adverse events were associated with the bronchoscopy procedures. This study was approved by the Institutional Ethics Committee of the Maternal and Child Health Hospital of Hubei Province [2022] IEC (018).

### Conventional microbiological tests

Routine samples were collected, including BALF, sputum, and blood. CMTs were performed within 2 days of admission, including sputum and BALF culture and smear (acid-fast staining for *Mycobacterium tuberculosis*; India ink staining for *Cryptococcus*), nasopharyngeal (NP) swab multiplex PCR (13 respiratory pathogens), BALF PCR (for *Mycoplasma pneumoniae*), serum antibody test (for *M. pneumoniae*), antigen test (for influenza virus A/B, 1,3-β-D-glucan antigen), and serum and BALF galactomannan test (*Aspergillus* spp.). The detection methods are specified in **Supplementary Tables 1** and **2**.

### Clinical comprehensive analysis was regarded as the reference standard

Based on the clinical diagnosis, two experienced clinicians analyzed all patients’ CMT and mNGS results, along with their medical records. First, each clinician determined whether the patient had pneumonia, based on the Chinese guidelines for the diagnosis of pneumonia in children (National Health Commission of the People’s Republic of China, State Administration of Traditional Chinese Medicine, 2019), according to clinical symptoms, pulmonary imaging, and clinical laboratory examination results. Second, etiology was determined by a comprehensive analysis of the patient’s clinical manifestations, laboratory findings, lung imaging, microbiological examination, and treatment response. If there was disagreement between clinicians, another senior clinician was consulted and a consensus was reached.

### Nucleic acid extraction, library preparation, and sequencing

Bronchoscopy was performed according to standard procedures using a flexible fiberoptic bronchoscope. A special collector was used to collect 3–5 ml of BALF, which was stored at 4°C. The BALF was sent for mNGS analysis (DNA and RNA). DNA was extracted using a QIAamp^®^ UCP Pathogen DNA Kit (Qiagen), following the manufacturer’s instructions. Human DNA was removed using benzonase (Qiagen) and Tween20 (Sigma). Total RNA was extracted using a QIAamp^®^ Viral RNA Kit (Qiagen). Ribosomal RNA was removed using a Ribo-Zero rRNA Removal Kit (Illumina). Complementary DNA (cDNA) was generated using reverse transcriptase and deoxynucleoside triphosphates (Thermo Fisher Scientific). Libraries were constructed for DNA and cDNA samples using the Nextera XT DNA Library Prep Kit (Illumina). Library quality was assessed using the Qubit dsDNA HS Assay Kit, followed by a high-sensitivity DNA Kit (Agilent) on an Agilent 2100 bioanalyzer. Library pools were then loaded onto an Illumina NextSeq CN500 sequencer for 75 cycles of single-end sequencing, generating approximately 20 million reads per library. For negative controls, we prepared peripheral blood mononuclear cell samples (10^5^ cells/ml) from healthy donors, in parallel with each batch, using the same protocol. Sterile deionized water was extracted alongside the specimens to serve as a non-template control.

### Bioinformatic analyses

Trimmomatic was used to remove low-quality reads, adapter contamination, duplicate reads, and reads shorter than 50 bp. Low-complexity reads were removed using K-complexity, with default parameters. Human sequence data were identified and excluded by mapping to a human reference genome (hg38) using Burrows–Wheeler Aligner software. We designed a set of criteria, similar to the criteria of the National Center for Biotechnology Information (NCBI), for selecting representative assemblies of microorganisms (bacteria, viruses, fungi, protozoa, and other multicellular eukaryotic pathogens) from the NCBI Nucleotide and Genome databases (National Center for Biotechnology Information). These were selected according to three references: (1) Johns Hopkins ABX Guide^1^; (2) Manual of Clinical Microbiology (Manual of clinical microbiology); and (3) case reports and research articles published in current peer-reviewed journals (Fiorini et al., 2017). The final database consisted of approximately 13,000 genomes. Microbial reads were aligned to the database using SNAP v1.0 beta 18 (Zaharia et al., 2021). Virus-positive detection results (DNA or RNA viruses) were defined by coverage of three or more non-overlapping regions in the genome. A positive detection was reported for a given species or genus when RMP was ≥5 or when RPM-r was ≥5. RPM-r was defined as RPM corresponding to a given species or genus in the clinical sample divided by RPM in the negative control (Miller et al., 2019). To minimize cross-species misalignments among closely related microorganisms, we discounted the RPM of a species or genus that appeared in non-template controls and shared a genus or family designation; a penalty of 5% was used for species (Zaharia et al., 2021).

### Statistical analysis

SPSS 19 (IBM Corporation) was used to perform all analyses. Clinical composite diagnosis and determination of microbiological etiology were regarded as reference standards. At the pathogen level, sensitivity, specificity, positive predictive value, negative predictive value, and accuracy were calculated using standard formulas for proportions. Wilson’s method was used to determine 95% confidence intervals for these proportions. McNemar’s test was used to compare diagnostic performance between CMTs and mNGS. All tests were two-tailed. A *p*-value of <0.05 was considered statistically significant.

Note that some children with multiple microbial infections had multiple class labels for this study (bacteria, viruses, fungi, and atypical pathogens). We report sensitivity, specificity, accuracy, and positive predictive value as performance measurements to permit direct comparisons between mNGS and CMTs.

## Results

### Patient characteristics

Among 594 eligible patients, 489 were excluded because they did not receive mNGS. Thus, we enrolled 103 (68 male and 35 female) patients. Their mean age was 4.5 years. Their clinical characteristics are shown in **Table 1**. The main clinical symptoms were as follows: fever, cough/sputum, wheezing, dyspnea, and hemoptysis. There were 22 cases (21.4%) admitted to an intensive care unit, 39 cases (38%) had recurrent respiratory infections, 54 cases (52.4%) had atelectasis/consolidation, 12 cases (11.7%) had pleural effusion, 4 cases (3.9%) had emphysema/mediastinum, and 6 cases (5.8%) had bronchiectasis. Bronchoscopy revealed 42 cases (40.8%) with poor ventilation, 8 cases (7.8%) with sputum embolus, 5 cases (4.9%) with bronchial mucosal necrosis, and 6 cases (5.8%) with bronchiectasis. All patients received empirical antibiotic therapy prior to admission.

**Table 1:** Clinical characteristics of 103 patients.

Patient characteristics	All patients (*n* = 103)
Age, years (mean ± SD)	4.5 ± 5.4
Male/Female	68 (66%)/35 (34%)
ICU [*n* (%)]	22 (21.4%)
Recurrent respiratory infection [*n* (%)]	40 (39%)
**Clinical symptoms**	
Fever	45 (43.7%)
Cough/sputum	74 (72.8%)
Wheezing	38 (36.9%)
Dyspnea	23 (22.3%)
Hemoptysis	2 (1.9%)
**Imaging features [*n* (%)]**	
Pulmonary consolidation/atelectasis	54 (52.4%)
Pleural effusion	12 (11.7%)
Mediastinal emphysema/emphysema	4 (3.9%)
Bronchiectasis	6 (5.8%)
**Bronchoscopy results [*n* (%)]**	
Poor ventilation	42 (40.8%)
Sputum emboli	8 (7.8%)
Mucosal necrosis	5 (4.9%)
Bronchiectasis	6 (5.8%)
**Laboratory examination [*n* (%)]**	
WBC >12 × 10^9^/L or <4 × 10^9^/L (normal 4.4–11.9 × 10^9^/L)	47 (45.6%)
CRP >10 mg/L (normal <4 mg/L)	13 (12.6%)
PCT >0.5 ng/ml (normal <0.5 ng/ml)	18 (17.5%)
D-dimer >0.5 μg/ml (normal <0.5 μg/ml)	43 (41.7%)
LDH > 300 U/L (normal < 300 U/L)	49 (47.6%)
Age, years (mean ± SD)	3.43 ± 3.65
Male/Female	70 (67%)/35 (33%)
ICU [*n* (%)]	22 (21%)
Recurrent respiratory infection [*n* (%)]	39 (37%)
**Clinical symptoms**	
Fever	45 (42.9%)
Cough/sputum	76 (72.4%)
Wheezing	38 (36.2%)
Dyspnea	23 (21.9%)
Hemoptysis	2 (1.9%)
**Imaging features [*n* (%)]**	
Pulmonary consolidation/atelectasis	56 (53.3%)
Pleural effusion	13 (12.4%)
Mediastinal emphysema/emphysema	4 (3.8%)
Bronchiectasis	6 (5.7%)
**Bronchoscopy results [*n* (%)]**	
Poor ventilation	42 (42.9%)
Sputum emboli	8 (7.2%)
Mucosal necrosis	5 (4.8%)
Bronchiectasis	6 (5.7%)
**Laboratory examination [*n* (%)]**	
WBC > 12 × 10^9^/L or <4 × 10^9^/L (normal 4.4–11.9 × 10^9^/L)	48 (45.7%)
CRP > 10 mg/L (normal < 4 mg/L)	13 (25.7%)
PCT > 0.5 ng/ml (normal < 0.5 ng/ml)	18 (17.1%)
D-dimer > 0.5 μg/ml (normal < 0.5 μg/ml)	44 (41.9%)
LDH > 300 U/L (normal < 300 U/L)	51 (48.6%)

WBC, white blood cell; CRP, C-reaction protein; PCT, procalcitonin; LDH, lactate dehydrogenase.

### Comparison of pathogen detection between CMTs and mNGS

**Figure 2:**
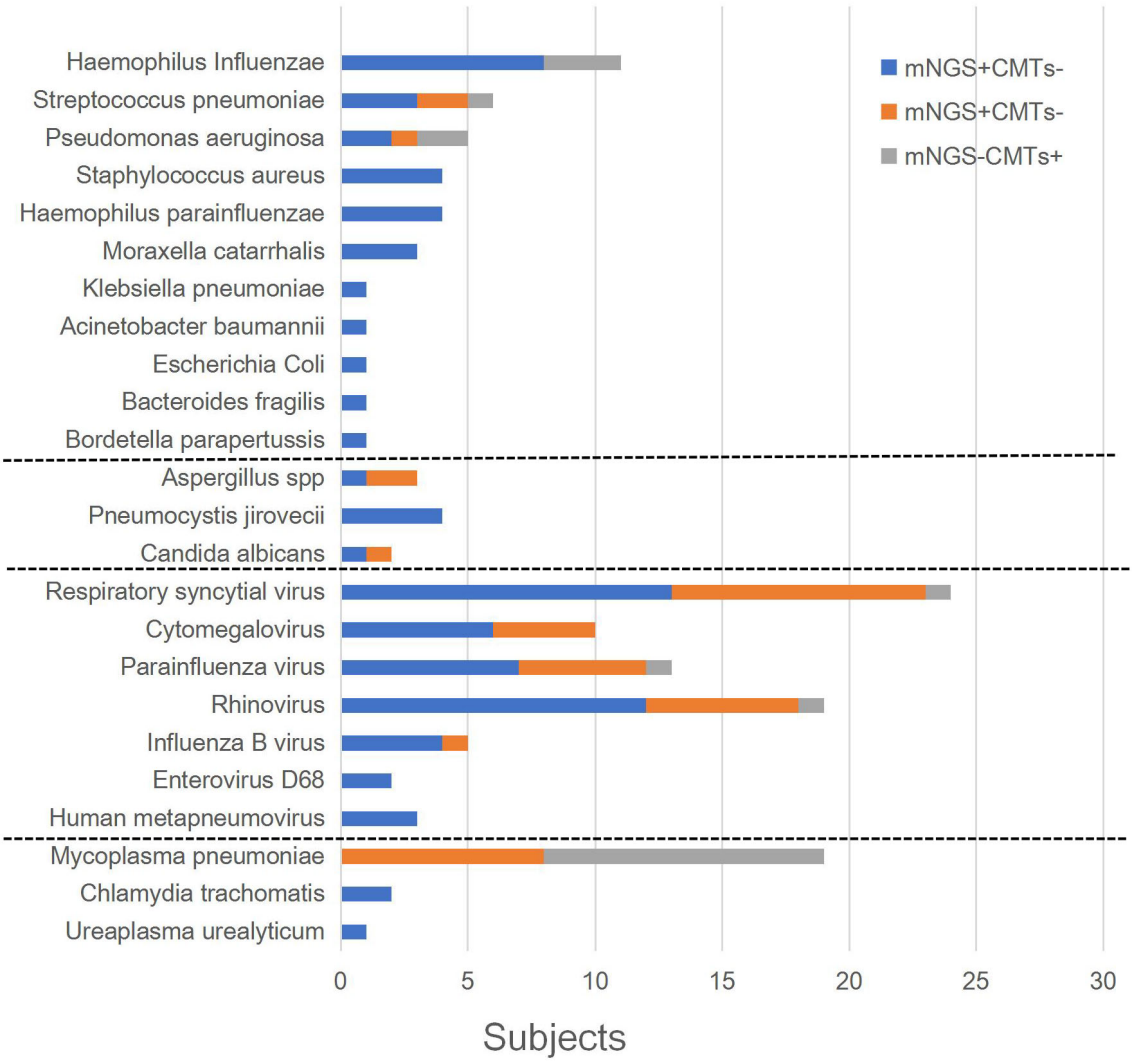
Distribution of pathogens identified by CMT versus mNGS.

The patients’ microbiological results are provided in **Supplementary Table 3**. Among them, 59.2% (61/103) cases tested positive using CMTs, and 91.3% (94/103) tested positive using mNGS (*p* = 0.000). In clinical comprehensive analysis, 93.2% (96/103) of patients had identified etiology. **Figure 2** shows the distribution of pathogens that met the definition of infection. Respiratory syncytial virus (26 cases), cytomegalovirus (25 cases), and parainfluenza virus (20 cases) were the top three viral infections. *Haemophilus influenzae* (12 cases), *Streptococcus pneumoniae* (6 cases), and *Pseudomonas aeruginosa* (6 cases) were the top three bacterial infections. *M. pneumoniae* (23 cases) was the most frequently detected atypical pathogen. Fungi were also identified, including *Aspergillus* spp., *Pneumocystis jirovecii*, and *Candida albicans*.

As shown in **Table 2**, there were 52 cases (50.5%) with monomicrobial infection and 44 cases (45.8%) with polymicrobial infection (30 cases were two-microbial infections, 13 cases were three-microbial infections, and 1 case was a four-microbial infection). There were seven cases with unidentified etiology (one patient was positive for *Circovirus* in blood mNGS but was not clinically considered to be infected, three cases were clinically considered to be viral pneumonia, and three cases were clinically considered to be bacterial pneumonia). Among 52 patients with monomicrobial infection, 27 cases (51.9%, 27/52) were detected using CMTs, while 48 cases (92.3%, 48/52) were detected using mNGS. Among 44 polymicrobial infections, 6 cases (13.6%, 6/44) were detected using CMTS, while 29 cases (65.9%, 29/44) were detected using mNGS. For single and mixed-microbial infections, the detection rate of mNGS was higher than that of CMTs (*p* = 0.000). The most common mixed infections were bacterial and viral. *Staphylococcus aureus*, *H. influenzae*, rhinovirus, cytomegalovirus, parainfluenza virus, and fungi were more likely to be associated with polymicrobial infections.

**Table 2:** Pneumonia pathogens in 96 patients.

	ALL (*n* = 96)	Monomicrobial (*n* = 52)	Polymicrobial (*n* = 44)	*p*-value
**Bacteria**	36 (37.5%)	12 (23%)	24 (55%)	0.005
* Streptococcus pneumoniae*	6 (6%)	1 (2%)	5 (11%)	0.021
* Staphylococcus aureus*	5 (5%)	2 (4%)	3 (7%)	0.527
* Haemophilus influenzae*	11 (11%)	1 (2%)	10 (23%)	0.000
Other bacteria	16 (17%)	8 (15%)^a^	8 (19%)^b^	1.000
**Virus**	67 (70%)	28 (54%)	39 (89%)	0.057
* Respiratory syncytial virus*	24(25%)	12(23%)	12 (27%)	1.000
* Rhinovirus*	20 (21%)	6 (12%)	14 (32%)	0.011
* Cytomegalovirus*	10 (10%)	0 (0%)	10 (23%)	0.000
* Influenza virus*	4 (4%)	2 (4%)	2 (5%)	1.000
* Parainfluenza virus*	13 (14%)	1 (2%)	12 (27%)	0.000
Other virus	12 (13%)	5 (10%)^c^	7 (16%)^d^	0.414
**Fungi**	9 (9%)	2 (4%)	7 (16%)	0.018
* Aspergillus* spp.	4 (4%)	1 (2%)	3 (7%)	0.157
* Pneumocystis jirovecii*	4 (4%)	1 (2%)	3 (7%)	0.157
Other fungi	2 (2%)	0 (0%)	2 (5%)^e^	0.046
**Atypical pathogen**	23 (24%)	12 (23%)	11 (25%)	0.768
* Mycoplasma pneumoniae*	19 (20%)	12 (23%)	7 (16%)	0.105
Other	3 (3%)	0 (0%)	3 (7%)^f^	0.014

a Including Pseudomonas aeruginosa (n = 2), Haemophilus parainfluenzae (n = 3), Bacteroides fragilis (n = 1), Escherichia coli (n = 1), and Bordetella parapertussis (n = 1).

b Including Acinetobacter baumannii (n = 1), Pseudomonas aeruginosa (n = 3), Haemophilus parainfluenzae (n = 1), Morella catarrhalis (n = 3), Klebsiella pneumoniae (n = 1), and Enterobacter cloacae complex (n = 1).

c Including Enterovirus D68 (n = 1), Human metapneumovirus (n = 1), and Bocavirus (n = 3).

d Including Human metapneumovirus (n = 2), Bocavirus (n = 4), and Enterovirus D68 (n = 1).

e Including Candida albicans (n = 2).

f Including Chlamydia trachomatis (n = 2) and Ureaplasma urealyticum (n = 1).

### Comparison of diagnostic performance between CMTs and mNGS

The diagnostic performance of CMTs and mNGS varied significantly among the different types of pathogens (**Table 3**). For bacterial detection, the diagnostic sensitivity (88.6% [78.0%–99.1%] vs. 25.7% [11.2%–40.1%], *p* < 0.001) and accuracy (87.4% [81.0%–93.8%] vs. 70.9% [62.1%–79.6%], *p* < 0.001) of mNGS were significantly higher than those of CMTs (95% confidence intervals shown). The positive predictive value (PPV) of mNGS was 77.5% (64.6%–90.4%) and the negative predictive value [NPV] was 93.7% (87.6%–99.7%). However, mNGS did not differ significantly from CMTs in the diagnosis of common bacterial infections, such as *S. pneumoniae* (*p* = 0.625), *S. aureus* (*p* = 0.219), and *H. influenza*e (*p* = 0.146). For virus detection, the sensitivity (100% [100%–100%]) and accuracy (86.4% [79.8%–93.2%]) of mNGS were slightly higher than those of CMTs (48.4% [36.2%–60.7%] and 68.0% [58.9%-77.0%], respectively). The PPV of mNGS was lower than that of CMTs (82.1% [73.5%–90.6%] vs. 100% [100%–100%]). The PPV of mNGS varied greatly for the different viruses. Apart from influenza virus (*p* = 0.125), the diagnostic performance of mNGS was significantly different from that of the CMTs (*p* < 0.01). For fungi detection, the diagnostic sensitivity of mNGS was higher than that of CMTS (87.5% [64.6%–100%] vs. 25.0% [0%–55%], *p* < 0.01) but the specificity (83.2% [75.6%–90.7%] vs. 97.9% [95.0%–100%]), accuracy (83.5% [76.3%–90.7%] vs. 92.2% [87.1%–97.4%]), and PPV (30.4% [11.6%–49.2%] vs. 50.0% [10.0%–99.0%]) were lower than those of CMTs. The detection sensitivity of mNGS for *P. jirovecii* was 100% (100%–100%), which could be verified by CMTs, such as hexamethyltetramine silver staining, implying a sensitivity of 0%. However, the PPV of mNGS for *P. jirovecii* was relatively low (33.3% [6.7%–60%]). For *Aspergillus* spp., the diagnostic performances of mNGS and CMTs were not significantly different (*p* = 0.219). The specificity and accuracy of both methods were higher than 96.1%; however, mNGS had higher sensitivity than CMTs (75% [32.6%–100%] vs. 50.0% [10%–99%]). There were no significant differences in the detection of atypical pathogens between mNGS and CMTs (*p* = 0.077). However, for *M. pneumoniae*, the sensitivity of mNGS was lower than that of CMTs (42.1% [20.0%–64.3%] vs. 100% [100%–100%], *p* = 0.001).

**Table 3:** Comparison of diagnostic performance of mNGS and CMTs.

	McNemar	mNGS					CMTs				
	NGS vs. CMTs, *p*	Sensitivity%(95% CI)	Specificity%(95% CI)	PPV%(95% CI)	NPV%(95% CI)	Accuracy(95% CI)	Sensitivity%(95% CI)	Specificity%(95% CI)	PPV%(95% CI)	NPV%(95% CI)	Accuracy%(95% CI)
**Bacteria**	0.000	88.6 (78.0–99.1)	86.8 (78.7–94.8)	77.5 (64.6–90.4)	93.7 (87.6–9.7)	87.4 (81.0–93.8)	25.7 (11.2–40.1)	94.1 (88.5–99.7)	69.2 (44.1–94.3)	71.1 (61.7–80.5)	70.9 (62.1–79.6)
* Streptococcus pneumoniae*	0.625	83.3 (53.5–100)	100 (100–100)	10 (100–100)	99.0 (97.0–100)	99.0 (97.1–100)	50.0 (10.0–90.0)	100 (100–100)	100 (100–100)	97.0 (93.7–100)	97.1 (93.8–100)
* Staphylococcus aureus*	0.219	100 (100–100)	100 (100–100)	100 (100–100)	100 (100–100)	100 (100–100)	0 (0–0)	96.0 (97.0–100)	0 (0–0)	95.81 (90.9–99.3)	94.2 (89.7–98.7)
* Haemophilus influenzae*	0.146	72.7 (46.4–99.0)	98.9 (96.8–100)	88.9 (68.4–100)	96.8 (93.2–100)	96.1 (92.4–99.8)	27.3 (1–53.6)	100 (100–100)	100 (100–100)	92.0 (86.7–97.3)	92.2 (87.1–97.4)
Other bacteria	0.001	87.5 (71.3–100)	87.4 (80.4–94.3)	56.0 (36.5–75.5)	97.4 (93.9–100)	87.4 (81.0–93.8)	18.8 (0–37.9)	95.4 (91.0–99.8)	42.9 (6.2–79.5)	86.5 (79.6–93.3)	83.5 (76.3–90.7)
**Virus**	0.000	100 (100–100)	64.1 (49.0–79.2)	82.1 (73.5–90.6)	100 (100–100)	86.4 (79.8–93.2)	48.4 (36.2–60.7)	100 (100–100)	100 (100–100)	54.2 (42.7–65.7)	68.0 (58.9–77.0)
Respiratory syncytial virus	0.001	95.8 (87.7–100)	98.7 (96.3–100)	95.8 (87.7–100)	98.7 (96.3–100)	98.1 (95.4–100)	45.8 (25.9–65.8)	100 (100–100)	100 (100–100)	85.9 (78.8–93.0)	87.4 (81.0–93.8)
Rhinovirus	0.002	94.7 (84.7–100)	96.4 (92.5–100)	85.7 (70.7–100)	98.8 (96.4–100)	96.1 (92.4–99.8)	36.8 (15.2–58.5)	98.8 (96.5–100)	87.5 (64.6–100)	87.4 (80.7–94.4)	87.4 (81.0–93.8)
Cytomegalovirus	0.000	100 (100–100)	84.9 (777–92.2)	41.7 (21.9–61.4)	100 (100–100)	86.4 (79.8–93.0)	40.0 (9.6–70.4)	100 (100–100)	100 (100–100)	93.9 (89.2–98.6)	94.2 (89.7–98.7)
Influenza virus	0.125	100 (100–100)	100 (100–100)	100 (100–100)	100 (100–100)	100 (100–100)	20.0 (0–55.1)	100 (100–100)	100 (100–100)	96.1 (92.3–99.8)	96.1 (92.4–99.8)
Parainfluenza virus	0.007	85.7 (56.2–97.5)	93.3 (88.0–98.5)	66.7 (44.9–88.4)	97.6 (94.4–100)	92.2 (87.1–97.4)	42.9 (16.9–68.8)	98.9 (96.7–100)	85.7 (59.8–100)	91.7 (86.1–97.2)	91.3 (85.8–96.7)
Other virus	0.000	90.9 (73.9–100)	75.0 (66.2–83.8)	30.3 (14.6–46.0)	98.6 (95.8–100)	76.7 (68.5–84.9)	45.5 (16.0–74.9)	100 (100–100)	100 (100–100)	93.9 (89.1–98.6)	94.2 (89.7–98.7)
**Fungi**	0.000	87.5 (64.6–100)	83.2 (75.6–90.7)	30.4 (11.6–49.2)	98.7 (96.3–100)	83.5 (76.3–90.7)	25.0 (0–55.0)	97.9 (95.0–100)	50.0 (10.0–99.0)	93.6 (89.2–98.6)	92.2 (87.1–97.4)
* Aspergillus* spp.	0.219	75.0 (32.6–100)	97.0 (93.6–100)	50 (10.0–90.0)	99.0 (97.0–100)	96.1 (92.4–99.8)	50.0 (10–99)	100 (100–100)	100 (100–100)	98.0 (95.3–100)	98.1 (95.3–100)
* Pneumocystis jirovecii*	NA	100 (100–100)	91.9 (86.6–97.3)	33.3 (6.7–60.0)	100 (100–100)	92.2 (87.1–97.4)	0 (0–0)	100 (100–100)	NA	96.1 (92.4–99.8)	96.1 (92.4–99.8)
Other fungi	0.250	NA	95.1 (91.0–99.3)	0 (0–0)	100 (100–100)	95.1 (91.0–99.3)	NA	98.1 (95.4–100)	0 (0–0)	100 (100–100)	98.1 (95.4–100)
**Atypical pathogen**	0.077	45.8 (25.9–65.8)	97.5 (94.0–100)	84.6 (65.0–100)	85.6 (78.3–92.8)	85.4 (78.6–92.2)	83.3 (68.4–98.2)	98.7 (96.3–100)	95.2 (86.1–100)	95.1 (90.5–99.8)	95.1 (91.0–99.3)
* Mycoplasma pneumoniae*	0.002	42.1 (20.0–64.3)	98.8 (96.5–100)	88.9 (68.4–100)	88.9 (68.4–100)	88.3 (88.2–94.5)	100 (100–100)	97.6 (94.4–100)	90.5 (77.9–100)	100 (100–100)	98.1 (95.4–100)
Other	NA	60.0 (17.1–100)	99.0 (97.0–100)	75.0 (32.6–100)	98.0 (95.2–100)	97.1 (93.8–100)	0 (0–0)	100 (100–100)	NA	95.1 (91.0–99.3)	95.1 (91.0–99.3)

CMTs, conventional microbiological tests; mNGS, metagenomics next-generation sequencing; NPV, negative predictive value; PPV, positive predictive value; CI, confidence interval.

## Discussion

Pneumonia is one of the most common causes of hospitalization for infection in children, and one of the most important causes of their morbidity and mortality (Liu et al., 2015). With extensive use of antibiotics, continuous expansion of the pathogen spectrum, and increasing numbers of hard-to-diagnose infections, it is increasingly difficult to identify the etiology of pneumonia. Relevant literature shows that comprehensive conventional methods do not find pathogens in up to 60% cases (Schlaberg et al., 2017). For patients with severe pneumonia, a long clinical course, the empirical use of antibiotics, and low immunity, CMTs are far from meeting the clinical need for etiology diagnosis; this may lead to the failure of therapy and the overuse of antibiotics. The bronchoalveolar lavage technique can be used to obtain cells and solutions from the lower respiratory tract. It is performed more easily and safely as the technique matures. In clinical practice, for patients with severe illness or suspected mixed infection, clinicians may examine several pathogens at the same time. However, they must verify these pathogens based on their own experience. By contrast, mNGS can detect all possible pathogens for clinicians’ judgment, which can save patients’ time and money. In recent years, BALF mNGS has become a breakthrough application for the diagnosis and treatment of infectious lung diseases.

mNGS has potential advantages in terms of speed and sensitivity for detecting lung diseases (Langelier et al., 2018; Li et al., 2018; Miao et al., 2018). Our study showed that, compared with CMTs, mNGS had a significant advantage in its detection rate of pathogens (91% vs. 59%, *p* = 0.000), even though all patients had used antibiotics. These results are consistent with the conclusions of Miao et al. (2018). mNGS was also superior to CMTs in diagnosing monomicrobial infections (92% vs. 52%, *p* = 0.000) and polymicrobial infections (66% vs. 14%, *p* = 0.000). Bacteria and viruses are pathogens commonly found in clinical settings. Our results showed that *S. aureus*, *H. influenzae*, rhinovirus, cytomegalovirus, parainfluenza virus, and fungi are more likely to be associated with polymicrobial infections, which suggests the advantages of mNGS in the diagnosis of mixed infections (Fang et al., 2020). Because mNGS can detect almost all microbes in BALF, the technique strongly support improvements in clinical intervention. Many studies (Wang et al., 2019a; Chen et al., 2021) have confirmed the superiority of mNGS in the diagnosis of pulmonary mixed infections and the identification of etiology.

A retrospective cohort study (Quah et al., 2018) found that the proportion of respiratory viruses in the pathogen spectrum of severe pneumonia has increased. In our study, 67 cases (70%) had viral infections, of which 28 cases were single infections and 39 were co-infections. Common viruses with high detection rates were respiratory syncytial virus, cytomegalovirus, rhinovirus, influenza virus, parainfluenza virus, and bocavirus. The sensitivity and accuracy of mNGS were higher than those of CMTs for the diagnosis of viral infections (**Table 3**). Owing to the difficulty of viral culture and the high rate of false positives in nucleic acid detection, it can be difficult to determine the etiology of the viruses identified (Ren et al., 2018). The relative abundance and read ratios of mNGS samples, relative to the negative control, may provide some clues for the determination of viral infections. However, in clinical practice, DNA testing alone may miss some RNA viruses, resulting in a decreased detection rate (Zhang et al., 2020). Messenger RNA of DNA viruses, detected in mNGS RNA testing, may provide clues regarding active transcription (Graf et al., 2016). Thus, performing both mNGS DNA and RNA testing is valuable in diagnosing the etiology of pneumonia. The unbiased nature of mNGS is useful for the detection of new and variant viruses (Chen et al., 2020), evolutionary tracing (Lu et al., 2020), and strain identification (Qian et al., 2021), as well as for guiding epidemiological investigations, public health research, and epidemic prevention and control during infectious disease outbreaks (Deurenberg et al., 2017). mNGS played a key role in the rapid identification of pathogens in the outbreak of the novel coronavirus pneumonia in late 2019 (Chen et al., 2020; Ren et al., 2020).

Our study revealed that the diagnostic performance of mNGS varied for different pathogens (**Table 3**). For detecting bacterial infections, the overall sensitivity (88.6% vs. 25.7%), accuracy (87.4% vs. 70.9%), PPV, and NPV of mNGS were higher than those of CMTs. However, there was no significant difference between mNGS and CMTs for the diagnosis of *S. pneumoniae*, *S. aureus*, and *H. influenzae* (*p* > 0.05). This differs slightly from previous studies (Xie et al., 2019) and may be related to the fact that these bacteria are clinically common in pediatric pneumonia, where empiric therapy is effective. mNGS has the advantage of being able to detect more pathogens. The sensitivity and accuracy of mNGS were higher than those of CMTs for the diagnosis of viral infection (*p* < 0.01); however, the PPV varied among different viruses. In particular, our study revealed that the parallel detection of DNA and RNA can determine the activity of DNA viruses and detect RNA viruses.

For fungal infections, the overall sensitivity of mNGS was higher than that of CMTs; however, the specificity and accuracy were lower than those of CMTs. The total NPV of mNGS was 98.7% (96.3%–100%). Positive results for *P. jirovecii*, such as staining microscopic examination and PCR, are important diagnostic criteria; however, the detection rates are low. *P. jirovecii* was detected in 12 patients using mNGS. In comprehensive clinical analysis, only four cases were considered to be pneumocystis pneumonia. These cases were all infants, in whom the course of disease was >2 weeks and the effect of conventional treatment was not good. This may be related to the fact that fungal infection was secondary to low immunity after infection. The remaining eight patients recovered without antifungal therapy; *P. jirovecii* was probably colonized in the lower respiratory tract. Unfortunately, our study was not further validated using Gomori methenamine silver staining, which may have influenced the comparison of the two testing methods. Recent studies (Wang et al., 2019b; Lin et al., 2022) have shown that mNGS has good diagnostic performance in the detection of pneumocystis. The identification of *Aspergillus* spp. by mNGS remains a challenge because of the difficulty of extracting DNA from its thick polysaccharide cell walls (Bittinger et al., 2014). Three cases of severe pneumonia with *Aspergillus* spp. etiology were reported by He et al. (2019). mNGS results indicated *Aspergillus* spp., and the patients were adjusted for antifungal treatment; their conditions improved. Thus, the accuracy of mNGS for the detection of *Aspergillus* spp. is suggested. In contrast with these results, our study did not show an advantage of mNGS for the diagnosis of *Aspergillus* infections. However, the small number of cases of fungal pneumonia in this study likely introduced biases in the calculation of diagnostic performance.

Although the specificity and accuracy of mNGS were high for *M. pneumoniae* diagnosis, the sensitivity was significantly lower than that of CMTs. In our study, mNGS did not show an advantage for the diagnosis of *M. pneumoniae* infections. The diagnosis of *M. pneumonia*e was confirmed by serology in the early stage of the disease (before admission to our hospital); the detection rate may have decreased after treatment. For the detection of *M. pneumoniae*, it has been reported that combined detection methods can improve the specificity and sensitivity of diagnosis and reduce false-negative and false-positive rates. *M. pneumoniae* cannot be reliably diagnosed using only a single test (Loens and Ieven, 2016; Tang et al., 2021).

Overall, mNGS can improve the detection rate of pathogens and mixed infections in pediatric pneumonia. The diagnostic utility of mNGS differs for different pathogens. For fungi and *M. pneumoniae*, the CMT approach may need to be combined to improve diagnostic performance. mNGS is valuable as a complement to CMTs, especially when the clinician does not have a presumed pathogen or the local laboratory is without complete CMTs.

This study has some limitations. First, the sample size was small, especially for fungal pneumonia. Second, *P. jirovecii* lacked further validation by Gomori methenamine silver staining; partial PCR failed to evaluate the diagnostic value of CMTs and mNGS. Third, at the time of this study, our hospital had had real-time PCR detection items for some pathogens, including 13 respiratory pathogens; there were no commercial offerings based on real-time multiplex PCR for the detection of community or hospital pathogens. Therefore, we did not compare the performances of multiplex PCR and mNGS for the detection of different pathogens. However, the use of real-time multiplex PCR assays is based on the clinician’s belief that a patient is infected with one or more of these pathogens; it ignores rare and unknown pathogens. Finally, the interpretation of mNGS results, to a certain extent, depended on the subjective judgment of the clinician, which may have led to bias.

## Data availability statement

The data presented in the study are deposited in the NCBI SRA repository, accession number SRR21425639~SRR21425741.

## Author contributions

JL: Designed the study and revised and approved the final version. WD: Drafted the initial manuscript, retrieved pediatric literature, and edited the table and reference list. YW: Participated in formal analysis. HX: Participated in data analysis. All authors contributed to the article and approved the submitted version.

## Acknowledgments

All the authors would like to express their appreciation to all the patients for their cooperation.

## Conflict of interest

The authors declare that the research was conducted in the absence of any commercial or financial relationships that could be construed as a potential conflict of interest.

## Publisher’s note

All claims expressed in this article are solely those of the authors and do not necessarily represent those of their affiliated organizations, or those of the publisher, the editors and the reviewers. Any product that may be evaluated in this article, or claim that may be made by its manufacturer, is not guaranteed or endorsed by the publisher.

## Footnotes

1
https://www.hopkinsguides.com/hopkins/index/Johns_Hopkins_ABX_Guide/Pathogens
